# Machine Learning-Based Prediction and Feature Attribution Analysis of Contrast-Associated Acute Kidney Injury in Patients with Acute Myocardial Infarction

**DOI:** 10.3390/medicina62010228

**Published:** 2026-01-22

**Authors:** Neriman Sıla Koç, Can Ozan Ulusoy, Berrak Itır Aylı, Yusuf Bozkurt Şahin, Veysel Ozan Tanık, Arzu Akgül, Ekrem Kara

**Affiliations:** 1 Division of Nephrology, Department of Internal Medicine, Ankara Etlik City Hospital, 06170 Ankara, Türkiye; silacank@hotmail.com (N.S.K.); arzuakgul@gmail.com (A.A.); 2 Perinatology Department, Bingöl State Hospital, 12000 Bingöl, Türkiye; canozanulusoy@gmail.com; 3 MSc Data Science Program, Hacettepe University, 06800 Ankara, Türkiye; 4 Department of Health Sciences, Hacettepe University, 06100 Ankara, Türkiye; itirayli@gmail.com; 5 Department of Cardiology, Ankara Etlik City Hospital, 06170 Ankara, Türkiye; ybozkurtsahin@gmail.com (Y.B.Ş.); drozantanik@gmail.com (V.O.T.); 6 Division of Nephrology, Department of Internal Medicine, Faculty of Medicine, Recep Tayyip Erdogan University, 53100 Rize, Türkiye

**Keywords:** contrast-associated acute kidney injury, acute myocardial infarction, machine learning, risk prediction, inflammation indices

## Abstract

*Background and Objectives*: Contrast-associated acute kidney injury (CA-AKI) is a frequent and clinically significant complication in patients with acute myocardial infarction (AMI) undergoing coronary angiography. Early and accurate risk stratification remains challenging with conventional models that rely on linear assumptions and limited variable integration. This study aimed to evaluate and compare the predictive performance of multiple machine learning (ML) algorithms with traditional logistic regression and the Mehran risk score for CA-AKI prediction and to explore key determinants of risk using explainable artificial intelligence methods. *Materials and Methods*: This retrospective, single-center study included 1741 patients with AMI who underwent coronary angiography. CA-AKI was defined according to KDIGO criteria. Multiple ML models, including gradient boosting machine (GBM), random forest (RF), XGBoost, support vector machine, elastic net, and standard logistic regression were developed using routinely available clinical and laboratory variables. A weighted ensemble model combining the best-performing algorithms was constructed. Model discrimination was assessed using area under the receiver operating characteristic curve (AUC), along with sensitivity, specificity, positive predictive value (PPV), and negative predictive value (NPV). Model interpretability was evaluated using feature importance and SHapley Additive exPlanations (SHAP). *Results*: CA-AKI occurred in 356 patients (20.4%). In multivariable logistic regression, lower left ventricular ejection fraction, higher contrast volume, lower sodium, lower hemoglobin, and higher neutrophil-to-lymphocyte ratio (NLR) were independently associated with CA-AKI. Among ML approaches, the weighted ensemble model demonstrated the highest discriminative performance (AUC 0.721), outperforming logistic regression and the Mehran risk score (AUC 0.608). Importantly, the ensemble model achieved a consistently high NPV (0.942), enabling reliable identification of low-risk patients. Explainability analyses revealed that inflammatory markers, particularly NLR, along with sodium, uric acid, baseline renal indices, and contrast burden, were the most influential predictors across models. *Conclusions*: In patients with AMI undergoing coronary angiography, interpretable ML models, especially ensemble and gradient boosting-based approaches, provide superior risk stratification for CA-AKI compared with conventional methods. The high negative predictive value highlights their clinical utility in safely identifying low-risk patients and supporting individualized, risk-adapted preventive strategies.

## 1. Introduction

Contrast-associated acute kidney injury (CA-AKI), defined as an acute decline in renal function following exposure to iodinated contrast media, remains a significant cause of hospital-acquired morbidity and mortality in modern medicine [1]. In line with current terminology, we use the term CA-AKI to reflect an association with contrast exposure rather than proven causality, particularly in AMI, where multiple hemodynamic and inflammatory contributors to AKI may coexist. With the increasing use of coronary, endovascular, and structural catheter-based interventions, CA-AKI has emerged as a clinically important complication associated with prolonged hospitalisation, increased healthcare costs, and adverse short- and long-term outcomes [2].

The development of CA-AKI is influenced by a multifactorial interplay of patient-related and procedure-related determinants. Among clinical risk factors, pre-existing chronic kidney disease, diabetes mellitus, advanced age, anemia, sepsis, and inadequate hydration are consistently identified as the strongest predictors of susceptibility to renal injury following contrast exposure [3]. Procedure-related contributors, such as the volume and osmolality of contrast media, repeated contrast exposure, and the presence of hemodynamic instability, further amplify the risk by exacerbating renal vasoconstriction and oxidative stress. These factors collectively contribute to renal hypoperfusion, tubular injury, and endothelial dysfunction, forming the central pathophysiological mechanisms underlying CA-AKI [3]. Therefore, patients undergoing percutaneous coronary interventions (PCI) or radiological imaging procedures represent a particularly vulnerable population in whom individualized risk stratification and preventive strategies are essential. A major limitation of current CA-AKI diagnosis is its reliance on serum creatinine, which is known to be a delayed and insensitive indicator of acute kidney injury, often rising only after substantial loss of renal function. Consequently, many early or subclinical injury phases remain undetected using creatinine-based criteria [4]. In this context, machine learning-based models that integrate routinely available clinical and laboratory data may function as an early warning system, enabling risk stratification well before overt creatinine-defined injury occurs [5].

Recent review evidence underscores that while conventional biomarkers such as serum creatinine lack sufficient sensitivity and timeliness, a broad array of emerging biomarkers (including Neutrophil Gelatinase-Associated Lipocalin (NGAL), cystatin C, hypoalbuminemia, hyperuricemia) demonstrate significant but heterogeneously validated predictive associations with CA-AKI [6].

Parallel to biomarker research, over the past few years, machine learning (ML)-based methods have shown great promise in predicting acute kidney injury and CA-AKI risks by leveraging patients’ individualized clinical and biochemical profiles [7,8]. Advanced algorithms, including Random Forest, Gradient Boosting, LightGBM, k-Nearest Neighbours, and multilayer perceptrons, have demonstrated promising predictive performance and, in some cohorts, improved discrimination compared with conventional statistical models [7,8,9,10,11]. However, many of these models were internally validated only, and relatively few have undergone external or multicenter validation, which limits direct comparison and generalizability. For example, Sun et al. achieved an area under the curve (AUC) of 0.82 in predicting CA-AKI among patients with acute myocardial infarction using the Random Forest algorithm, substantially outperforming traditional logistic regression models [8]. Similarly, Lim et al. developed a successful prediction model for CA-AKI, achieving an AUC of 0.914 by using only preoperative variables in patients undergoing lower-extremity perfusion angioplasty procedures [10].

Despite these encouraging findings, most existing ML-based CA-AKI prediction models have been developed within relatively narrow or procedure-specific cohorts, and often focus primarily on predictive performance [7,12]. Limited attention has been paid to comprehensive model comparison, clinical interpretability, or benchmarking against established risk scores, all of which are critical for translation into routine clinical practice [9,13]. Moreover, the integration of readily available inflammatory indices and systematic explainability analyses remains insufficiently explored in real-world AMI populations [7,12,13].

This study aims to address the need for an accurate and clinically interpretable prediction of CA-AKI in patients with acute myocardial infarction (AMI) undergoing coronary angiography. To this end, clinical, biochemical, and demographic variables were comprehensively evaluated, and traditional statistical methods were integrated with ML-based approaches. In addition to logistic regression, advanced ML algorithms were employed to identify independent determinants of CA-AKI, develop and internally validate ML-based classification models for individualized risk prediction, and enhance model interpretability through SHapley Additive exPlanations (SHAP)-based feature attribution analyses. This holistic approach aims to bridge the gap between conventional risk scoring and data-driven clinical decision-support systems, thereby contributing both methodologically and clinically to early CA-AKI risk stratification and the development of personalized preventive strategies.

## 2. Materials and Methods

### 2.1. Study Design, Patient Cohort, and Data Collection

This retrospective observational study aimed to predict CA-AKI in patients who underwent coronary angiography due to acute myocardial infarction, comprising ST-elevation MI or non-ST-elevation MI, between January 2020 and June 2025. The study was conducted at a single center and included data from a total of 1741 patients. All eligible cases were retrospectively reviewed using the hospital’s electronic medical records. Patients with cardiogenic shock requiring inotropes or intra-aortic balloon pump support were excluded to avoid confounding from hemodynamic-driven acute kidney injury, thereby allowing the model to focus on contrast-associated renal injury patterns. This population restriction may limit generalizability and may also influence comparability with risk scores that incorporate severe hemodynamic variables (e.g., the Mehran score). Patients on maintenance hemodialysis or with a history of kidney transplantation, individuals with clinical evidence of active infection, and those with missing laboratory or clinical data were also excluded from the study.

CA-AKI was defined according to serum creatinine component of the Kidney Disease: Improving Global Outcomes (KDIGO) criteria as any of the following after contrast exposure: an increase in serum creatinine to ≥1.5 times the baseline within 7 days or an increase in serum creatinine by ≥0.3 mg/dL within 48 h [14]. Urine output criteria were not applied because urine output measurements were not consistently available in this retrospective dataset.

All patients received the same non-ionic, low-osmolar contrast material iohexol with a measured osmolality of 844 mosm/kg H_2_O (Omnipaque 350; GE Healthcare, Princeton, NJ, USA). For all patients, demographic, clinical, biochemical, and hematological parameters on admission were systematically collected.

Formal automated feature selection algorithms (e.g., Boruta) were not applied; instead, model inputs were determined based on clinical relevance, prior literature, data availability at presentation, and univariable screening, with additional assessment of multicollinearity to avoid redundant predictors. This approach was chosen to preserve clinically interpretable and routinely accessible features. The collected data were categorised into three main groups:

- Demographic and clinical parameters: age, gender, body mass index (BMI), presence of comorbidities, current status of smoking and alcohol consumption, pre-existing chronic use of medication at the time of angiography, systolic and diastolic blood pressure, mean arterial pressure (MAP), heart rate, left ventricular ejection fraction, and procedural data (contrast volume and revascularization status), type of AMI symptom (atypical or typical chest pain), Mehran Score, Killip Classification for Heart Failure.
- Laboratory parameters: Pre-angiography hematologic indices, plasma glucose, creatinine, estimated glomerular filtration rate (eGFR), blood urea nitrogen (BUN), albumin, uric acid, C-reactive protein (CRP), electrolytes, lipid profile.
- Derived inflammatory indices: Neutrophil-to-lymphocyte (NLR) and platelet-to-lymphocyte ratios (PLR), CRP/albumin ratio (CAR) were calculated.

#### 2.1.1. Risk Score and Clinical Variable Calculations

##### Mehran Risk Score

The risk of CA-AKI after PCI was estimated using the original Mehran risk score model described by Mehran et al. [15], which assigns weighted integers to eight clinical and procedural variables based on their independent association with CA-AKI. These variables include hypotension (systolic blood pressure < 80 mm Hg requiring inotropic support), intra-aortic balloon pump use, congestive heart failure, age > 75 years, anemia, diabetes mellitus, total contrast volume, and baseline renal function status. The sum of the weighted points yields an individual patient risk score for CA-AKI. Patients were subsequently stratified into four predefined risk categories based on their total score. These categories reflect the estimated risk of CA-AKI:

- Low risk (1): ≤5 points
- Moderate risk (2): 6–10 points
- High risk (3): 11–15 points
- Very high risk (4): ≥16 points

##### Killip Classification

Heart failure severity at presentation was classified according to the Killip–Kimball classification, first described by Killip and Kimball in 1967, in patients with acute myocardial infarction [16]. Patients were categorized into four classes based on clinical findings: Class I (no signs of heart failure), Class II (mild heart failure with rales, S3 gallop or elevated jugular venous pressure), Class III (pulmonary edema), and Class IV (cardiogenic shock). This clinical stratification has been widely validated as a predictor of adverse outcomes following acute coronary syndromes.

Derived inflammatory, anthropometric, and hemodynamic indices were calculated using standard formulas as follows:

MAP = (Systolic Blood Pressure + 2 × Diastolic Blood Pressure)/3

CAR = C-reactive Protein (mg/dL)/Serum Albumin (g/dL)

BMI = Weight (kg)/Height (m)^2^

NLR = Neutrophil (10^3^/µL)/Lymphocyte (10^3^/µL)

PLR = Platelet (10^3^/µL)/Lymphocyte (10^3^/µL)

### 2.2. Statistical Analysis

Statistical analyses were performed using R (version 4.5.1) within R Studio (version 2025.01.0) utilizing the caret, pROC, xgboost, randomForest, gbm, e1071, glmnet, and SHAPforxgboost packages.

Continuous variables were assessed for normality using visual inspection of histograms and the Shapiro–Wilk test. As most continuous variables were not normally distributed, they are presented as median (interquartile range [IQR]) and were compared between groups using the Mann–Whitney U test. Categorical variables are expressed as number (percentage) and were compared using the chi-square test or Fisher’s exact test, as appropriate.

Univariate logistic regression analyses were performed to identify variables associated with the development of CA-AKI. Variables with clinical relevance and/or a *p* value < 0.10 in univariate analysis were entered into a multivariable logistic regression model using the enter method. Multicollinearity was assessed prior to model construction, and correlated variables were not entered simultaneously into the same model. Results are reported as odds ratios (ORs) with 95% confidence intervals (CIs). Model discrimination was evaluated using the area under the receiver operating characteristic curve (AUC).

The Mehran risk score and Mehran risk categories were analyzed separately as reference models and were not included in the multivariable regression to avoid redundancy.

### 2.3. Machine Learning Analysis

Machine learning-based models were developed to predict CA-AKI. The following algorithms were evaluated: (1) elastic net logistic regression, (2) random forest (RF), (3) gradient boosting machine (GBM), (4) extreme gradient boosting (XGBoost), (5) support vector machine (SVM) with radial basis function kernel, and (6) standard logistic regression.

Data were randomly split into training (80%) and test (20%) sets using stratified sampling to preserve outcome distribution. Continuous variables were standardized, and categorical variables were one-hot encoded using a preprocessing pipeline. No synthetic oversampling (SMOTE) was applied in order to preserve the original outcome distribution. Feature standardization and one-hot encoding were fitted exclusively on the training set and subsequently applied to the test set using the same parameters, in order to prevent potential data leakage. Feature selection procedures, including near-zero variance detection and high-correlation removal (|r| > 0.90), were conducted solely on training data, with the resulting feature set applied to both datasets.

Hyperparameter tuning was performed using 10-fold cross-validation repeated 3 times for caret-based models (elastic net, random forest, GBM, SVM, logistic regression), optimizing the area under the AUC. For XGBoost, 5-fold cross-validation with early stopping (30 rounds) was used to prevent overfitting. Final hyperparameters are reported in Supplementary Table S2.

Multicollinearity among predictor variables was assessed using variance inflation factors (VIF) calculated from a logistic regression model fitted to training data. Variables with VIF > 10 were considered to exhibit substantial multicollinearity. Additionally, pairwise Pearson correlation coefficients were computed, and one variable from each pair with |r| > 0.90 was removed to reduce redundancy.

### 2.4. Ensemble Learning

A weighted ensemble model was constructed by combining predictions from the three best-performing individual models (GBM, random forest, and XGBoost). Model weights were proportional to each model’s cross-validated AUC, calculated as:

$$P_{ensemble}=\;\frac{(w\_(GBM)\cdot P\_(GBM)\;+\;w\_(RF)\cdot P\_(RF)\;+\;w\_(XGB)\cdot P\_(XGB))\;}{\left(w\_(GBM)\;+\;w\_(RF)\;+\;w\_(XGB)\right)}$$

where *P* represents predicted probabilities and *w* represents the cross-validated AUC for each respective model.

### 2.5. Model Evaluation

Model performance was evaluated on the independent test set using the following metrics: AUC with 95% CIs estimated using the DeLong method, sensitivity, specificity, positive predictive value (PPV), negative predictive value (NPV), and F1 score. Optimal classification thresholds were determined using Youden’s J statistic (sensitivity + specificity − 1). The discriminative ability of ML models was compared against the Mehran risk score, a validated clinical risk score for CA-AKI prediction. Statistical comparison of AUCs was performed using the DeLong test. Model calibration was assessed using the Brier score and Hosmer–Lemeshow goodness-of-fit test. Calibration plots were constructed by dividing predicted probabilities into deciles and comparing mean predicted versus observed event rates within each group. A Hosmer–Lemeshow *p* value > 0.05 was considered indicative of acceptable calibration.

### 2.6. Model Interpretability

Model interpretability was evaluated using model-specific variable importance measures and Shapley additive explanations (SHAP). For the GBM, variable importance was quantified using relative influence, defined as the total reduction in squared error attributable to each predictor across all trees. For the RF model, importance was assessed using mean decrease in Gini impurity. For the XGBoost model, feature importance was derived from information gain.

In addition, SHAP values were computed for the XGBoost model to provide both global and local explanations. Global importance was summarized using mean absolute SHAP values, while local explanations quantified the direction and magnitude of each feature’s contribution to individual predictions. SHAP summary plots were generated to visualize the distribution of feature effects across the study population.

### 2.7. Statistical Significance

A two-sided *p* value < 0.05 was considered statistically significant for all analyses. All statistical tests were performed using R (version 4.5.1) within RStudio (version 2025.01.0), and figures were generated using ggplot2 and base R graphics. 

## 3. Results

### 3.1. Baseline Characteristics and Group Comparisons

A total of 1741 patients who required coronary angiography due to AMI were included in the analysis, of whom 356 (20.4%) developed CA-AKI. Patients who developed CA-AKI were significantly older than those without CA-AKI (median age 62 vs. 59 years, *p* < 0.001). The distribution of gender, BMI, smoking status, alcohol consumption, and comorbidities was comparable between groups. Antiplatelet therapy, angiotensin-converting enzyme inhibitor use, and statin therapy were less frequent in patients who developed CA-AKI (Table 1).

Myocardial infarction symptom type and hemodynamic parameters at presentation were similar between groups. However, patients with CA-AKI had a higher Mehran risk score (median 5.0 vs. 4.0, *p* < 0.001) and were more frequently classified into higher Mehran risk categories (*p* < 0.001). Left ventricular ejection fraction was significantly lower in the CA-AKI group (median 48% vs. 50%, *p* < 0.001). Although median contrast volume was similar, the upper distribution was higher in patients who developed CA-AKI (*p* < 0.001). Revascularisation procedures were performed more frequently in the CA-AKI group (82.6% vs. 76.6%, *p* = 0.016) (Table 2).

Patients who developed CA-AKI exhibited lower hemoglobin, lower lymphocyte count, lower triglycerides, lower sodium, and lower serum albumin levels, along with higher inflammatory and metabolic markers, including WBC, CRP, CAR, NLR, PLR, plasma glucose, serum uric acid, blood urea nitrogen and contrast/eGFR ratio (all *p* < 0.001). Baseline eGFR was modestly but significantly lower in the CA-AKI group, whereas baseline serum creatinine and glycated hemoglobin (HbA1c) levels did not differ significantly (Table 3).

### 3.2. Univariable and Multivariable Logistic Regression

In univariate analysis, older age, lower left ventricular ejection fraction, status of revascularization performed, higher contrast volume, lower hemoglobin, lower albumin, lower sodium, lower baseline eGFR, higher NLR and PLR, higher WBC, higher CRP, CAR and higher contrast/eGFR ratio were associated with CA-AKI. In multivariable analysis, left ventricular ejection fraction (aOR 0.985 per 1% increase, *p* = 0.043), contrast volume (aOR 1.002 per mL, *p* = 0.019), hemoglobin (aOR 0.905 per g/dL, *p* = 0.025), sodium (aOR 0.918 per meq/L, *p* < 0.001), and NLR (aOR 1.051, *p* = 0.033) remained independently associated with CA-AKI. Status of revascularization performed, baseline eGFR, CAR, contrast/eGFR ratio and age lost statistical significance after adjustment. The Mehran risk score showed significant univariate association with CA-AKI but was analyzed separately as a reference model (Table 4).

### 3.3. Model Performance and Explainability

To predict the risk of CA-AKI, six machine learning classifiers (GBM, XGBoost, RF, SVM, standard logistic regression, and elastic net–regularized logistic regression) were developed and evaluated. In addition, a weighted ensemble model combining the three best-performing classifiers (GBM, random forest, and XGBoost) was constructed. Model performance was compared with the Mehran risk score. The study utilized a dataset comprising 1741 patients who underwent coronary angiography due to AMI, including clinical, biochemical, and demographic variables. The dataset was randomly divided into training (80%) and testing (20%) subsets.

**Figure 1:**
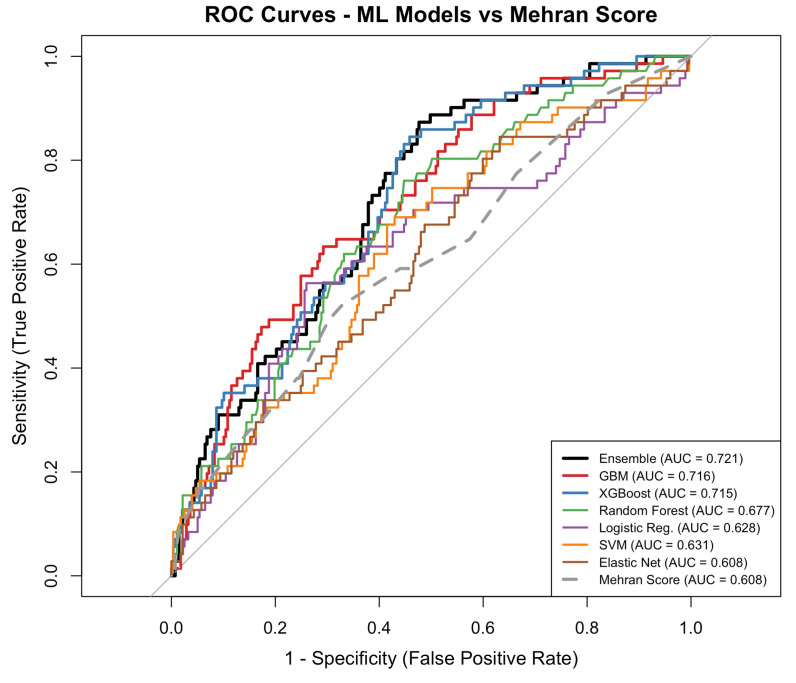
Receiver operating characteristic (ROC) curves of machine learning models for the prediction of contrast-associated acute kidney injury in the test cohort.

The performance of each model was evaluated using accuracy, precision, recall, F1-score, and AUC metrics. The obtained test results are summarized in Table 5 and illustrated in Figure 1. Model calibration was assessed using Brier Scores and the Hosmer–Lemeshow test (Table S1). The GBM model achieved the lowest Brier score (0.150) with a non-significant Hosmer–Lemeshow test (χ^2^ = 9.14, *p* = 0.330). The ensemble model showed acceptable calibration (Brier score = 0.153, Hosmer–Lemeshow *p* = 0.058). Calibration plots illustrated predicted and observed CA-AKI rates across the range of predicted risks (Figure S1).

### 3.4. ROC Curve Analysis

The ROC curves provide a comprehensive assessment of the models’ ability to distinguish between CA-AKI and non-CA-AKI cases.

Among machine learning models, the weighted ensemble model, fitted with XGBoost, GBM and random forest, demonstrated the highest discrimination, with a test-set AUC of 0.721 (95% CI, 0.659–0.782). This was followed by GBM (AUC 0.716; 95% CI, 0.652–0.780) and XGBoost (AUC 0.715; 95% CI, 0.653–0.777). Random forest showed lower discrimination (AUC 0.677; 95% CI, 0.610–0.745), while support vector machine, logistic regression, and elastic net models yielded AUC values ranging from 0.609 to 0.631. The Mehran score demonstrated an AUC of 0.608 (95% CI, 0.533–0.684).

At optimal thresholds determined by Youden’s index, sensitivity ranged from 0.521 for the Mehran score to 0.873 for the ensemble model, whereas specificity ranged from 0.368 for elastic net to 0.740 for logistic regression. Positive predictive values were modest across models (0.255–0.357), while negative predictive values were consistently high (0.845–0.942). F1 scores ranged from 0.372 to 0.468, with the highest value observed for the ensemble model (Table 5, Figure 1). The ensemble model demonstrated significantly better discrimination compared to the Mehran score (DeLong test *p* = 0.010), representing an 18.5% relative improvement in predictive performance.

### 3.5. Feature Importance and Explainability Analysis

**Figure 2:**
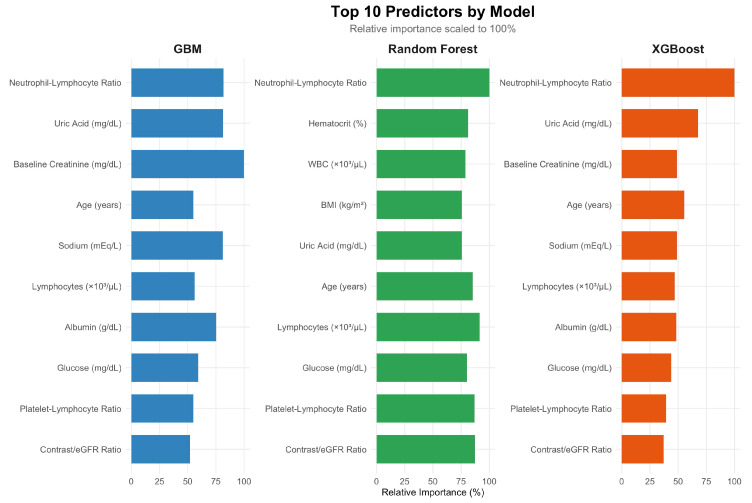
Top predictors of contrast-associated acute kidney injury across machine learning models. Top 10 predictors of contrast-associated acute kidney injury identified by three machine learning models: Gradient Boosting Machine, Random Forest, and XGBoost. Feature importance is shown as relative importance scaled to 100% within each model. Across all models, inflammatory markers, particularly NLR, consistently ranked among the most influential predictors, alongside baseline creatinine, metabolic parameters (uric acid, glucose), electrolyte status (sodium), and demographic factors (age). Differences in relative ranking reflect model-specific learning mechanisms, while overall concordance highlights shared clinically relevant risk patterns.

Variable importance rankings derived from tree-based models are presented in Figure 2. In the GBM, the highest relative importance was observed for NLR, followed by uric acid, baseline creatinine, sodium, age, lymphocyte count, albumin, glucose, PLR, and contrast-to-eGFR ratio. In the RF model, NLR ranked highest, followed by uric acid, sodium, antiplatelet use, CRP, albumin, age, lymphocyte count, and contrast-to-eGFR ratio. Importance values were scaled within each model to facilitate comparison of relative contributions.

### 3.6. SHAP Analysis

**Figure 3:**
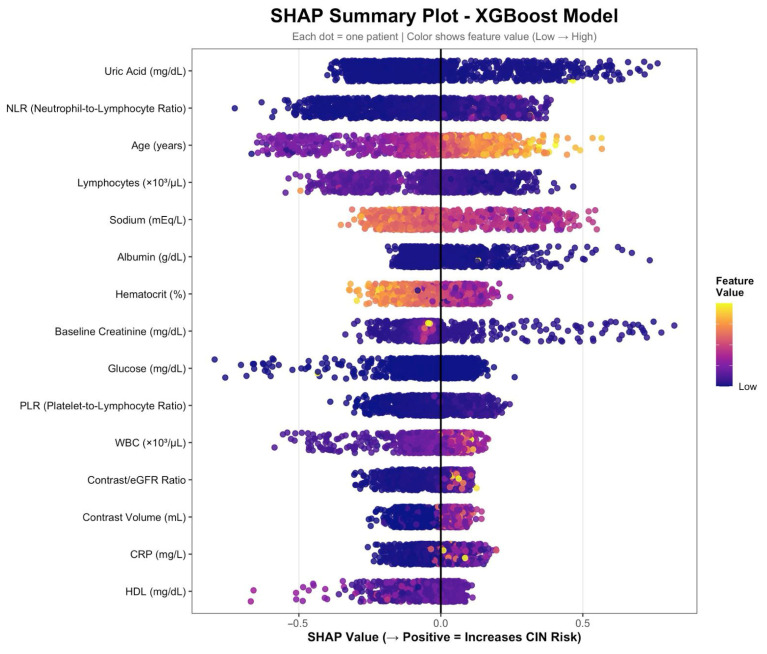
SHAP summary plot illustrating the relative importance and direction of clinical variables for the prediction of contrast-associated acute kidney injury using the XGBoost Model. SHAP (Shapley Additive Explanations) summary plot showing the contribution of each variable to the XGBoost model output for contrast-associated acute kidney injury prediction. Each dot represents an individual patient. The *x*-axis shows the SHAP value, indicating the impact of each variable on the predicted risk of contrast-associated acute kidney injury, with positive values increasing and negative values decreasing the predicted risk. Variables are ordered by decreasing mean absolute SHAP value. Colors represent the magnitude of the corresponding feature value (dark blue = low, yellow = high).

SHAP summary results for the XGBoost model are shown in Figure 3. Features with the largest absolute SHAP values included NLR, uric acid, sodium, age, lymphocyte count, contrast/eGFR ratio, albumin, PLR, hematocrit, glucose, baseline creatinine, CRP, contrast volume, and HDL. Each point represents an individual observation, with SHAP values indicating the magnitude and direction of each feature’s contribution to the model output. Feature values are color-coded to reflect their relative magnitude within the dataset.

## 4. Discussion

In this study, we evaluated the predictive performance of multiple machine learning models for contrast-associated acute kidney injury in patients with AMI undergoing coronary angiography and compared these approaches with traditional risk assessment strategies. Our findings suggest that several machine learning classifiers, particularly gradient boosting-based models and random forest, achieved improved discriminative performance compared with conventional logistic regression and the Mehran risk score. Importantly, beyond discrimination metrics, the best-performing models demonstrated consistently high NPV, supporting their potential utility as decision-support tools to help identify low-risk patients for CA-AKI.

Clinically, CA-AKI remains a frequent complication after contrast exposure, and it is strongly linked to worse short- and long-term outcomes, including prolonged hospitalisation and increased mortality, which makes peri-procedural risk stratification particularly important [1,6]. In our study evaluating 1741 patients who underwent coronary angiography due to AMI, 20.4% of them were found to have CA-AKI. In the present cohort, patients who developed CA-AKI were characterised by a distinct clinical and biochemical profile. In multivariable logistic regression analysis, left ventricular ejection fraction, contrast volume, sodium, hemoglobin, and NLR emerged as independent predictors of CA-AKI, whereas age and baseline eGFR lost statistical significance after adjustment. Consistent with previous clinical evidence, our findings emphasize that functional cardiac reserve, systemic inflammation, and hematologic vulnerability play a more prominent role in CA-AKI development than isolated demographic characteristics or baseline renal function measures in patients undergoing coronary angiography [1,6,17].

Mechanistically, contrast exposure superimposed on the hemodynamic and oxidative stress milieu of AMI may trigger renal medullary hypoxia, endothelial dysfunction, and tubular injury; inflammatory activation appears to amplify these pathways [18]. In this context, inflammation-related parameters such as CRP, CAR, NLR and PLR have been repeatedly explored as practical predictors of CA-AKI in cardiovascular interventions, echoing our observation that inflammation-derived features remain informative in risk prediction [19,20,21,22].

In this present study, the NLR emerged as the most influential predictor across both regression and machine learning analyses, underscoring the central role of systemic inflammation in CA-AKI pathogenesis. Physiological stress and inflammation induce catecholamine-mediated neutrophilia with concomitant lymphopenia. An elevated NLR therefore reflects a proinflammatory state that may aggravate renal injury through oxidative stress and immune dysregulation [23]. Consistent with our findings, a recent meta-analysis demonstrated that elevated NLR was associated with a 1.52-fold increased risk of acute kidney injury, supporting its robust prognostic relevance [24].

In our study, serum sodium emerged as a clinically and analytically robust predictor of CA-AKI, remaining independently associated with risk in multivariable regression and ranking among the most influential features in machine learning and SHAP-based analyses. Hyponatremia should not be viewed solely as an electrolyte disturbance, but rather as a marker of impaired effective circulating volume and neurohormonal activation. In acute myocardial infarction, the fall in sodium concentrations is closely related to activation of the sympathetic nervous system and the renin–angiotensin–aldosterone system, together with neurohormone-mediated, non-osmotic release of vasopressin [25]. Elevated vasopressin activity promotes renal water reabsorption and increases metabolic and oxygen demand in the renal medulla, a region intrinsically vulnerable to hypoxia [26]. When superimposed on contrast-induced renal vasoconstriction, this hemodynamic and hormonal milieu may further exacerbate medullary ischemia and thereby increase susceptibility to CA-AKI.

Although serum uric acid did not retain independent significance in multivariable regression, its importance in SHAP analysis suggests a biologically meaningful contribution to CA-AKI risk. Experimental and clinical data indicate that hyperuricemia inhibits endothelial nitric oxide synthase, leading to reduced nitric oxide bioavailability and enhanced renal vasoconstriction. In addition, uric acid promotes oxidative stress and microvascular inflammation, mechanisms that may synergistically amplify contrast-induced reductions in renal blood flow, particularly in susceptible patients [27].

The Mehran risk score remains one of the most widely used and validated tools for CA-AKI risk assessment in clinical practice [15]. Importantly, the Mehran score incorporates several procedural and peri-procedural variables, such as contrast volume and intra-procedural hemodynamic factors, which may restrict its utility for purely pre-procedural risk estimation. In contrast, machine learning models can integrate a broader range of baseline clinical and laboratory parameters, including inflammatory markers, enabling more flexible and individualized risk prediction before contrast exposure. In our study, machine learning models demonstrated higher discriminative performance compared with the Mehran score, albeit with a moderate overall improvement. This finding suggests that data-driven approaches may better accommodate the multifactorial and non-linear nature of CA-AKI risk.

In recent years, multiple machine learning-based models have been proposed to predict CA-AKI across heterogeneous clinical settings, including acute coronary syndromes, peripheral vascular interventions, emergency department contrast-enhanced imaging, and PCI [8,9,10]. These studies consistently demonstrate that ML algorithms can achieve modest to good discriminative performance, often comparable to or slightly exceeding conventional logistic regression or established risk scores such as the Mehran score [8,9,10]. However, model performance varies substantially depending on patient population, feature selection, and outcome definition, underscoring the importance of contextualizing ML outputs within the underlying clinical phenotype rather than focusing solely on absolute AUC values.

In our machine learning analyses, ensemble and gradient boosting-based models demonstrated higher discrimination for the prediction of contrast-associated acute kidney injury compared with conventional approaches. Among all models, the weighted ensemble classifier achieved the highest overall performance, with a test-set AUC of 0.721, followed closely by gradient boosting machine and XGBoost models. In contrast, traditional logistic regression and elastic net–regularized logistic regression yielded more modest discriminative ability, comparable to that of the Mehran risk score. This is consistent with contemporary studies reporting that ensemble-based gradient boosting algorithms outperform linear models in clinical prediction of CA-AKI and related renal outcomes [7,8].

Although the observed improvement in AUC was moderate, the absolute AUC improvement of the ensemble model over the Mehran score was statistically significant (DeLong *p* = 0.01) and the consistent performance advantage of machine learning models suggests that they better capture the complex, non-linear relationships underlying CA-AKI risk in patients with AMI. Feature importance and SHAP analyses further revealed that inflammatory markers, particularly the neutrophil-to-lymphocyte ratio, along with baseline renal function, metabolic parameters (uric acid and glucose), electrolyte status (sodium), and contrast burden, emerged as the most influential predictors across models. Notably, these results indicate that machine learning-based algorithms integrate multidimensional clinical information more effectively than rule-based risk scores, which may partly explain their superior predictive performance in this high-risk population. These findings are in line with prior explainable machine learning studies showing that CA-AKI prediction is driven by a combination of renal reserve, systemic inflammation, metabolic stress, and procedural exposure, rather than by any single isolated variable [7,8,9]. Furthermore, ML models are inherently adaptable and can be recalibrated or expanded to incorporate novel biomarkers, longitudinal data, or dynamic clinical variables without the need to redesign fixed scoring systems. In this respect, ML-based frameworks should be viewed not as replacements for validated clinical scores, but as flexible, explainable extensions that enhance mechanistic insight and support personalized preventive strategies.

Compared with prior ML studies that focused on heterogeneous elective coronary cohorts, CKD-only populations, or non-coronary contrast exposures, our AMI-restricted cohort may better preserve the acute hemodynamic–inflammatory signature relevant to CA-AKI pathogenesis [10,11,28,29]. While some AMI studies reported higher AUCs, differences in event incidence, feature engineering/selection, and validation strategies complicate direct comparisons [8]; importantly, our real-world AMI dataset with true prevalence and an independent test set demonstrates that an interpretable ensemble framework can provide clinically meaningful discrimination and outperform a traditional risk score in this unstable population. Therefore, model performance should not be interpreted solely through discrimination metrics, but also through clinically actionable measures such as negative predictive value, which may better support bedside decision-making in AMI.

Beyond overall discrimination metrics, the weighted ensemble model demonstrated a consistently high negative predictive value, which carries clinical relevance. A high NPV indicates that patients classified as low risk by the model are unlikely to develop contrast-associated acute kidney injury, allowing clinicians to confidently identify individuals in whom aggressive preventive strategies or intensive monitoring may be safely de-escalated. In the setting of acute myocardial infarction, where rapid decision-making and resource allocation are critical, this characteristic may be more clinically actionable than modest differences in AUC alone. Accordingly, the strength of the ensemble model lies not only in risk prediction, but also in its potential to support safe exclusion of CA-AKI in low-risk patients.

This feature is particularly valuable in routine practice, as it may help avoid unnecessary hydration protocols, nephrology consultations, or contrast avoidance strategies in patients with genuinely low risk.

### Limitations

Nevertheless, several limitations should be acknowledged. A first limitation of the present study relates to the exclusion of patients with severe pre-procedural hemodynamic instability (inotrope requirement and/or intra-aortic balloon pump support) to reduce etiologic confounding, since AKI in this subgroup is often predominantly shock-related rather than contrast-associated. However, this approach may limit real-world generalizability to the sickest AMI population and could disadvantage the Mehran score, because hemodynamic compromise is embedded in its components, thereby making the ML-versus-Mehran comparison more conservative. In addition, the comparison with the Mehran risk score should be interpreted with caution, as patients with severe hemodynamic compromise (including IABP use and hypotension requiring inotropes) were excluded, potentially attenuating the predictive contribution of key Mehran components and disadvantaging its performance in this cohort.

Second, the retrospective single-center design may introduce selection bias and limit causal inference. Moreover, given the complex hemodynamic milieu of AMI, KDIGO-defined AKI events occurring after angiography cannot be unequivocally attributed to contrast exposure alone and should be interpreted as post-procedural AKI associated with contrast exposure rather than proven contrast-induced nephropathy. Third, the absence of external or multicenter validation limits generalizability; therefore, performance may not directly translate to other AMI populations or healthcare settings. Accordingly, the ensemble model should be considered hypothesis-generating and not yet ready for routine clinical deployment until validated in independent multicenter cohorts. Fourth, the unavailability of certain emerging kidney injury biomarkers, such as neutrophil gelatinase–associated lipocalin and cystatin C, precluded their incorporation into the predictive models, which may have further enhanced discriminative performance [30]. Finally, despite rigorous internal validation with cross-validation, the risk of model overfitting cannot be entirely excluded, particularly for complex algorithms like XGBoost. Given these limitations, the present model should be considered hypothesis-generating rather than ready for clinical deployment, and prospective validation is warranted before implementation in routine practice.

Future research should aim to validate these findings in prospective, multicenter settings and to incorporate longitudinal renal outcomes together with multimodal biomarkers, including metabolomic and imaging data, into flexible and explainable machine learning frameworks. Such approaches may facilitate more precise risk stratification and enable proactive, patient-centered nephroprotective strategies in individuals undergoing contrast-based procedures.

In summary, interpretable ensemble learning may support individualized CA-AKI risk assessment and guide proactive nephroprotective strategies in AMI patients undergoing coronary angiography.

## 5. Conclusions

In a real-world cohort of patients with acute myocardial infarction undergoing coronary angiography, ML models, particularly gradient boosting-based approaches and a weighted ensemble classifier, demonstrated improved discrimination for predicting CA-AKI compared with conventional logistic regression and the Mehran risk score. Importantly, beyond risk prediction, the proposed ML framework has the potential to function as a clinical decision-support tool that enables personalized preventive strategies. Explainability analyses (feature importance and SHAP) highlighted a consistent set of clinically plausible drivers, including impaired cardiac reserve, contrast burden, electrolyte status, anemia, and systemic inflammation (NLR), supporting both predictive utility and mechanistic interpretability. At the same time, the consistently high NPV of the ensemble model allows reliable identification of low-risk patients who are unlikely to develop CA-AKI, facilitating safe de-escalation of preventive interventions and more efficient, patient-centered use of healthcare resources. Collectively, these findings suggest that interpretable ensemble-based machine learning approaches may advance precision medicine by enabling risk-adapted CA-AKI prevention in patients undergoing coronary angiography.

## Figures and Tables

**Table 1:** Baseline demographic characteristics, comorbidities, and medication use in patients with and without CA-AKI.

	CA-AKI (+) Group *n* = 356	CA-AKI (−) Group *n* = 1385	*p* Value
**Demographics**, median (IQR)—*n* (%)			
**Age**, years	62.0 (53.0, 71.0)	59.0 (51.0, 67.0)	<*0.001*
**Gender**			0.857
Female	88 (24.7)	336 (24.3)	
Male	268 (75.3)	1049 (75.7)	
**BMI**, kg/m^2^	27.9 (25.4, 31.2)	28.3 (25.7, 31.4)	0.494
**Current smoking**	252 (71.2)	977 (70.6)	0.666
**Alcohol consumption**	39 (11.1)	136 (9.9)	0.784
**Comorbidities**, *n* (%)			
**Hypertension**	179 (51.3)	707 (52.4)	0.699
**Diabetes mellitus**	116 (32.6)	442 (31.9)	0.809
**Asthma**	9 (2.5)	48 (3.5)	0.622
**COPD**	17 (4.8)	64 (4.6)	0.906
**Obstructive sleep apnea**	8 (2.2)	21 (1.5)	0.338
**Pulmonary arterial hypertension**	15 (4.2)	72 (5.2)	0.444
**Cerebrovascular disease**	22 (6.2)	52 (3.8)	0.057
**Chronic atrial fibrillation**	8 (2.2)	27 (2.0)	0.368
**Chronic heart failure**	7 (2.0)	24 (1.7)	0.768
**Chronic kidney disease**	18 (5.1)	74 (5.3)	0.829
**Medication**, *n* (%)			
**Antiplatelet therapy**	79 (22.3)	390 (28.2)	*0.025*
**ACE inhibitor use**	82 (23.2)	399 (28.9)	*0.032*
**Beta-blocker use**	69 (19.5)	318 (23.0)	0.154
**Statin therapy**	30 (8.5)	211 (15.3)	<*0.001*

Data are presented as median (IQR) or number (percentage), as appropriate. Continuous variables were compared using the Mann–Whitney U test, and categorical variables were compared using the chi-square test or Fisher’s exact test, as appropriate. A two-sided *p* value < 0.05 was considered statistically significant. ACE: angiotensin-converting enzyme; BMI: body mass index; CA-AKI: contrast-associated acute kidney injury; COPD: chronic obstructive pulmonary disease; IQR: interquartile range. Statistically significant values (*p* < 0.05) are italicized.

**Table 2:** Cardiac clinical presentation, risk scores, vital signs, and coronary angiography characteristics in patients with coronary artery disease with and without contrast-associated acute kidney injury.

	CA-AKI (+) Group *n* = 356	CA-AKI (−) Group *n* = 1385	*p* Value
**Cardiac Clinical Presentation & Scores**, median (IQR)—*n* (%)			
**MI Symptom type**			0.909
Typical	266 (78.7)	1006 (78.6)	
Atypical	72 (21.3)	275 (21.5)	
**Killip class**			0.073
Class 1	316 (92.4)	1244 (95.8)	
Class 2	7 (2.0)	12 (0.9)	
Class 3	14 (4.1)	33 (2.5)	
Class 4	5 (1.5)	10 (0.8)	
**Mehran risk category**			<*0.001*
Low risk (1)	205 (57.6)	977 (70.5)	
Moderate risk (2)	124 (34.8)	346 (25.0)	
High risk (3)	24 (6.7)	57 (4.1)	
Very high risk (4)	3 (0.8)	5 (0.4)	
**Mehran risk score**	5.0 (2.5–7.0)	4.0 (2.0–6.0)	<*0.001*
**Vitals**, median (IQR)			
**SBP, mmHg**	140.0 (121.0–160.0)	140.0 (123.0–160.0)	0.989
**DBP, mmHg**	80.0 (70.0–90.0)	80.0 (70.0–91.0)	0.626
**MAP, mmHg**	100.0 (87.5–113.0)	101.0 (90.0–113.0)	0.860
**Heart rate, beats/min**	78 (66–93)	80 (69–94)	0.218
**LVEF, %**	48.0 (40.0–55.0)	50.0 (43.0–60.0)	<*0.001*
**CAG**, median (IQR)—*n* (%)			
**Contrast volume, mL**	200.0 (100.0–300.0)	200.0 (100.0–250.0)	<*0.001*
**Revascularization performed**	294 (82.6)	1060 (76.6)	*0.016*

Data are presented as median (IQR) or number (percentage), as appropriate. Continuous variables were compared using the Mann–Whitney U test, and categorical variables were compared using the chi-square test or Fisher’s exact test, as appropriate. A two-sided *p* value < 0.05 was considered statistically significant. CAG: coronary angiography; CA-AKI: contrast-associated acute kidney injury; DBP: Diastolic blood pressure; IQR: interquartile range; MAP: Mean arterial pressure; MI: myocardial infarction; LVEF: Left ventricular ejection fraction; SBP: Systolic blood pressure. Statistically significant values (*p* < 0.05) are italicized.

**Table 3:** Baseline laboratory parameters in patients with coronary artery disease with and without contrast-associated acute kidney injury.

Variables, Median (IQR)	CA-AKI (+) Group *n* = 356	CA-AKI (−) Group *n* = 1385	*p* Value
**Hemoglobin**, g/dL	13.5 (11.9–14.7)	14.1 (12.8–15.2)	<*0.001*
**WBC count**, ×10^3^/µL	10.9 (8.4–13.5)	10.1 (8.2–12.5)	<*0.001*
**Platelet**, ×10^3^/µL	224 (190–267)	231 (195–272)	0.247
**Lymphocytes**, ×10^3^/µL	1.67 (1.19–2.3)	2.2 (1.56–3)	<*0.001*
**Plasma glucose**, mg/dL	134 (110–179)	121 (102–170)	<*0.001*
**HbA1c**, %	6.10 (5.70–7.38)	6.00 (5.70–7.10)	0.615
**Uric acid**, mg/dL	6.50 (5.20–7.90)	5.90 (5.00–7.00)	<*0.001*
**Sodium**, mEq/L	137 (135–140)	139 (137–141)	<*0.001*
**Potassium**, mEq/L	4.2 (3.9–4.6)	4.3 (4–4.6)	0.188
**BUN**, mg/dL	17.0 (14.0–23.8)	16.0 (13.0–20.0)	<*0.001*
**Baseline serum creatinine**, mg/dL	0.87 (0.75–1.16)	0.85 (0.77–1.03)	0.073
**Baseline eGFR**, mL/min/1.73 m^2^	90.2 (63.8–104.0)	94.8 (76.4–105.0)	*0.003*
**Triglycerides**, mg/dL	138 (94–190)	144 (104–211.25)	*0.017*
**Serum albumin**, g/dL	3.70 (3.50–4.00)	3.80 (3.70–4.10)	<*0.001*
**C-reactive protein**, mg/dL	1.20 (0.40–3.60)	0.80 (0.30–2.40)	<*0.001*
**CAR**	0.33 (0.12–1.02)	0.20 (0.07–0.65)	<*0.001*
**NLR**	5 (2.65–8.87)	2.95 (1.83–5.52)	<*0.001*
**PLR**	128.88 (88.74–197.52)	98.59 (70.88–143.02)	<*0.001*
**Contrast/eGFR ratio**	2.37 (1.7–3.2)	1.95 (1.2–2.8)	<*0.001*

Data are presented as median (interquartile range). Continuous variables were compared using the Mann–Whitney U test. A two-sided *p* value < 0.05 was considered statistically significant. BUN: blood urea nitrogen; CAR: C-reactive Protein/Serum Albumin ratio; CA-AKI: contrast-associated acute kidney injury; CRP: C-reactive protein; eGFR: estimated glomerular filtration rate; HbA1c: glycated hemoglobin; IQR: interquartile range; NLR: Neutrophil/Lymphocyte Ratio; PLR: Platelet/Lymphocyte Ratio; WBC: white blood cell count. Statistically significant values (*p* < 0.05) are italicized.

**Table 4:** Univariate and multivariate logistic regression analyses for predictors of contrast-associated acute kidney injury, with comparison to the Mehran risk score.

	Univariate LR		Multivariate LR		AUC
	OR (95% CI)	*p* Value	aOR (95% CI)	*p* Value	
**Age**	1.021 (1.011–1.031)	<*0.001*	1.010 (0.995–1.025)	0.192	
**Diabetes mellitus**	1.031 (0.804–1.322)	0.809			
**Chronic kidney disease**	0.943 (0.556–1.601)	0.829			
**Chronic heart failure**	1.137 (0.486–2.659)	0.768			
**MAP, mmHg**	1.000 (0.993–1.006)	0.921			
**LVEF, %**	0.973 (0.962–0.985)	<*0.001*	0.985 (0.970–1.000)	*0.043*	
**Contrast volume**, mL	1.002 (1.001–1.004)	<*0.001*	1.002 (1.000–1.004)	*0.019*	
**Revascularization performed**	1.445 (1.069–1.952)	*0.017*	1.143 (0.746–1.749)	0.540	
**Hemoglobin**, g/dL	0.867 (0.818–0.919)	<*0.001*	0.905 (0.830–0.988)	*0.025*	
**WBC count**, ×10^3^/µL	1.051 (1.024–1.078)	<*0.001*	0.997 (0.957–1.039)	0.884	
**Platelet**, ×10^3^/µL	0.999 (0.997–1.000)	*0.141*			
**Baseline eGFR**, mL/min/1.73 m^2^	0.990 (0.986–0.995)	<*0.001*	0.997 (0.989–1.006)	0.506	
**Uric acid**, mg/dL	0.999 (0.992–1.006)	0.776			
**Sodium**, mEq/L	0.877 (0.846–0.910)	<*0.001*	0.918 (0.878–0.959)	<*0.001*	
**Potassium**, mEq/L	0.935 (0.745–1.173)	0.559			
**Serum albumin**, g/dL	0.556 (0.412–0.750)	<*0.001* *			
**CRP**, mg/dL	1.045 (1.018–1.074)	<*0.001* *			
**CAR**	1.197 (1.088–1.317)	<*0.001*	1.126 (0.989–1.281)	0.072	
**NLR**	1.044 (1.024–1.064)	<*0.001*	1.051 (1.004–1.100)	*0.033*	
**PLR**	1.001 (1.000–1.001)	*0.04*	0.998 (0.997–1.000)	0.079	
**Contrast/eGFR ratio**	1.099 (1.053–1.148)	<*0.001*	0.981 (0.895–1.075)	0.676	
					0.628
**MEHRAN**					
**MEHRAN score**	1.109 (1.070–1.149)	<*0.001*			0.608
**MEHRAN categories**					0.607
2 vs. 1	1.716 (1.331–2.214)	<*0.001*			
3 vs. 1	2.017 (1.223–3.325)	*0.004*			
4 vs. 1	3.831 (1.020–14.391)	*0.047*			

Univariate and multivariable logistic regression analyses were performed to identify predictors of contrast-associated acute kidney injury. Results are presented as odds ratios (ORs) and adjusted odds ratios (aORs) with 95% confidence intervals (CIs). Variables with clinical relevance and/or *p* < 0.10 in univariate analysis were entered into the multivariable model. The Mehran risk score and Mehran risk categories were analyzed separately as reference models and were not included in the multivariable regression to avoid multicollinearity. Model discrimination was assessed using the AUC. A two-sided *p* value < 0.05 was considered statistically significant. * To minimize multicollinearity, when the CAR was included in the multivariable model, its component variables (CRP and serum albumin) were not entered simultaneously. aOR: adjusted odds ratio; AUC: area under the receiver operating characteristic curve; CAR: C-reactive Protein/Serum Albumin ratio; CI: confidence interval; CRP: C-reactive protein; eGFR: estimated glomerular filtration rate; LR: Logistic regression; LVEF: left ventricular ejection fraction; MAP: mean arterial pressure; OR: odds ratio; WBC: white blood cell. Statistically significant values (*p* < 0.05) are italicized.

**Table 5:** Performance of machine learning models for the prediction of contrast-associated acute kidney injury.

Model	AUC	95% CI	Sensitivity	Specificity	PPV	NPV	F1
**Ensemble (Weighted)**	0.721	0.659–0.782	0.873	0.523	0.320	0.942	0.468
**GBM**	0.716	0.652–0.780	0.634	0.708	0.357	0.883	0.457
**XGBoost**	0.715	0.653–0.777	0.845	0.542	0.321	0.932	0.465
**Random Forest**	0.677	0.610–0.745	0.761	0.552	0.303	0.900	0.434
**SVM**	0.631	0.558–0.703	0.676	0.585	0.294	0.876	0.410
**Elastic Net (logistic regression)**	0.609	0.535–0.682	0.845	0.368	0.255	0.903	0.392
**Logistic Regression**	0.628	0.550–0.705	0.563	0.740	0.357	0.869	0.437
**Mehran Score**	0.608	0.533–0.684	0.521	0.671	0.289	0.845	0.372

AUCs were calculated on the independent test set. AUC: area under the receiver operating characteristic curve; CI: confidence interval; PPV: positive predictive value; NPV: negative predictive value.

## Data Availability

The datasets used during the study have not been made publicly available due to patient privacy but are available from the corresponding author upon reasonable request.

## Supplementary Materials

The following supporting information can be downloaded at: https://www.mdpi.com/article/10.3390/medicina62010228/s1, Table S1. Model Calibration Assessment. Table S2. Optimal Hyperparameters for Machine Learning Models. Figure S1. Calibration Plots for the Top 4 Models.

## Abbreviations

The following abbreviations are used in this manuscript:

AMI	Acute Myocardial Infarction
AKI	Acute Kidney Injury
CA-AKI	Contrast-Associated Acute Kidney Injury
ACE	Angiotensin-Converting Enzyme
AUC	Area Under the Curve
BMI	Body Mass Index
BUN	Blood Urea Nitrogen
CAR	C-reactive Protein/Albumin Ratio
CI	Confidence Interval
COPD	Chronic Obstructive Pulmonary Disease
CRP	C-reactive Protein
DBP	Diastolic Blood Pressure
eGFR	Estimated Glomerular Filtration Rate
EF	Ejection Fraction
GBM	Gradient Boosting Machine
HbA1c	Glycated Hemoglobin
IABP	Intra-Aortic Balloon Pump
IQR	Interquartile Range
KDIGO	Kidney Disease: Improving Global Outcomes
LR	Logistic Regression
LVEF	Left Ventricular Ejection Fraction
MAP	Mean Arterial Pressure
ML	Machine Learning
MI	Myocardial Infarction
NLR	Neutrophil-to-Lymphocyte Ratio
NPV	Negative Predictive Value
OR	Odds Ratio
PCI	Percutaneous Coronary Intervention
PLR	Platelet-to-Lymphocyte Ratio
PPV	Positive Predictive Value
RF	Random Forest
ROC	Receiver Operating Characteristic
SBP	Systolic Blood Pressure
SHAP	SHapley Additive exPlanations
SVM	Support Vector Machine
WBC	White Blood Cell
XGBoost	Extreme Gradient Boosting
