# Supplementary material for: Machine Learning-Based Prediction and Feature Attribution Analysis of Contrast-Associated Acute Kidney Injury in Patients with Acute Myocardial Infarction
Source: Medicina (Kaunas). 2026 Jan 22;62(1):228. doi: 10.3390/medicina62010228 (PMC12844072; doi:10.3390/medicina62010228)
Supplement: Supplementary file 1 [file medicina-62-00228-s001.zip › medicina-4095557-supplementary.pdf]

SUPPLEMENTS

Table S1. Model Calibration Assessment

Model	Brier Score	Hosmer-Lemeshow χ^2	p-value
**GBM**	0.150	9.14	0.330
**Random Forest**	0.152	11.00	0.202
**Ensemble**	0.153	15.06	0.058
**Elastic Net**	0.159	4.40	0.819
**Logistic Regression**	0.161	25.60	0.001
**SVM**	0.162	12.61	0.126
**XGBoost**	0.153	12.70	0.079

Brier score ranges from 0 (perfect) to 1 (worst); lower values indicate better calibration. The Hosmer–Lemeshow test assesses goodness-of-fit; $p > 0.05$ indicates acceptable calibration. Calibration interpretation: Good ($p > 0.10$), Acceptable ($0.05 \leq p \leq 0.10$), Poor ($p < 0.05$).

Table S2. Optimal Hyperparameters for Machine Learning Models

Model	Parameter	Optimal Value
Logistic Regression	Family	Binomial
Elastic Net	Alpha (L1/L2 ratio)	1.0 (LASSO)
Elastic Net	Lambda (regularization)	0.0149
Random Forest	mtry (variables per split)	2
Random Forest	ntree (number of trees)	1000
GBM	n.trees	500
GBM	interaction.depth	6
GBM	shrinkage (learning rate)	0.01
GBM	n.minobsinnode	5
XGBoost	nrounds (iterations)	100
XGBoost	max_depth	4
XGBoost	eta (learning rate)	0.05
XGBoost	Subsample	0.8
XGBoost	colsample_bytree	0.8
XGBoost	scale_pos_weight	3.89

SVM	sigma (RBF kernel)	0.05
SVM	C (cost parameter)	0.25

Hyperparameters were optimized using 10-fold cross-validation repeated 3 times on the training set. XGBoost used 5-fold cross-validation with early stopping. No resampling techniques were used; class imbalance was handled implicitly by means of threshold optimization and performance metrics.

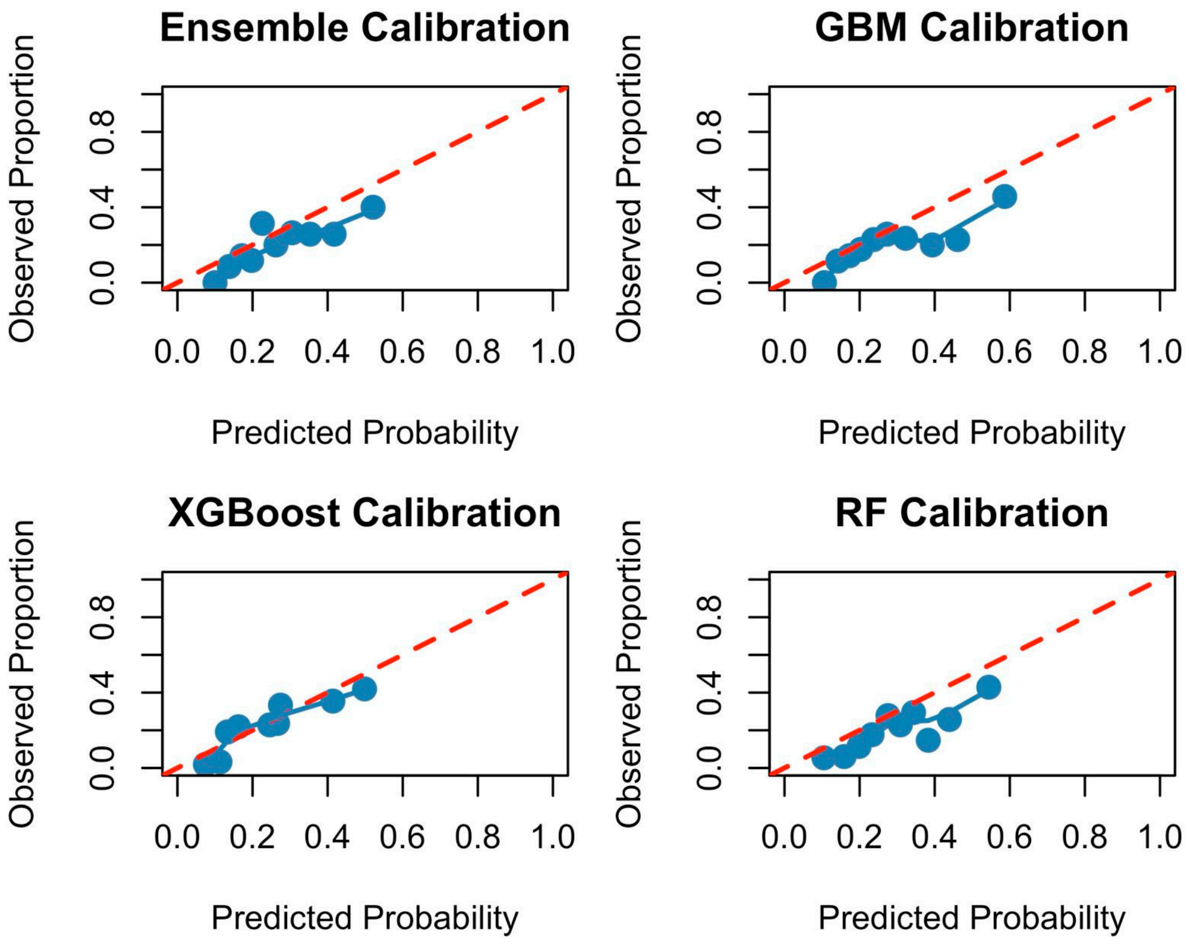

Figure S1. Calibration Plots for the Top 4 Models.
